# Quantity yields quality when it comes to creativity: a brain and behavioral test of the equal-odds rule

**DOI:** 10.3389/fpsyg.2015.00864

**Published:** 2015-06-25

**Authors:** Rex E. Jung, Christopher J. Wertz, Christine A. Meadows, Sephira G. Ryman, Andrei A. Vakhtin, Ranee A. Flores

**Affiliations:** ^1^Department of Psychology, University of New MexicoAlbuquerque, NM, USA; ^2^Department of Neurosurgery, University of New MexicoAlbuquerque, NM, USA

**Keywords:** creativity, creative cognition, divergent thinking, imagination, cortical volume, neuroimaging (anatomic and functional), magnetic resonance imaging

## Abstract

The creativity research community is in search of a viable cognitive measure providing support for behavioral observations that higher ideational output is often associated with higher creativity (known as the equal-odds rule). One such measure has included divergent thinking: the production of many examples or uses for a common or single object or image. We sought to test the equal-odds rule using a measure of divergent thinking, and applied the consensual assessment technique to determine creative responses as opposed to merely original responses. We also sought to determine structural brain correlates of both ideational fluency and ideational creativity. Two-hundred forty-six subjects were subjected to a broad battery of behavioral measures, including a core measure of divergent thinking (Foresight), and measures of intelligence, creative achievement, and personality (i.e., Openness to Experience). Cortical thickness and subcortical volumes (e.g., thalamus) were measured using automated techniques (FreeSurfer). We found that higher number of responses on the divergent thinking task was significantly associated with higher creativity (*r* = 0.73) as independently assessed by three judges. Moreover, we found that creativity was predicted by cortical thickness in regions including the left frontal pole and left parahippocampal gyrus. These results support the equal-odds rule, and provide neuronal evidence implicating brain regions involved with “thinking about the future” and “extracting future prospects.”

## Introduction

There is a long history, within the creativity literature, noting an association between idea fluency (the number of ideas generated) and the associated quality, originality, and/or creativity of the ideas that are produced on divergent thinking tasks (Wallach and Kogan, 1965). This notion has since been conceptualized as the “equal-odds rule” by Simonton (1997), which states that “the relationship between the number of hits (i.e., creative successes) and the total number of works produced in a given time period is positive, linear, stochastic, and stable.” This principle has great appeal in that it conforms broadly to evolutionary principles (i.e., there is a variation/selection process; Campbell, 1960), it is parsimonious (Simonton, 1984b), and it conforms to excitatory and inhibitory neuronal processes familiar to the neurosciences (Logothetis, 2008). However, this concept of productivity *leading to* originality is rarely exploited within either psychometric or neuroimaging studies of creative cognition, with most studies focused on rather convolved and/or abstruse psychometric aspects of creativity including (but not limited to) fluency, cognitive control, latent inhibition, improvisation, remote associates, divergent thinking, and the like (Arden et al., 2010).

As we have noted previously (Jung et al., 2013), the varieties of cognitive processes critical to creative cognition are likely to be relatively few when deconvolved from more general functions such as attention, memory, language, visual, spatial, and executive processes subserving most aspects of higher cognitive functioning. Variation/selection mechanisms facilitated by ideational fluency/quality presents a viable candidate for such a core cognitive and neuronal mechanism. Substantial support has been generated through historiometric analyses of Big C creative individuals, with the vast majority of individuals studied conforming to the equal-odds rule (Simonton, 1977, 1984a, 1985, 1988, 2010). The relationship is not universally observed, however, with one recent study of eminent composers finding a linear relationship between “hits” and age, which should be independent if conforming to “Darwinian” processes of variation/selection as opposed to accumulated experience (Kozbelt, 2008). Kozbelt and Ostrofsky (2013) further hypothesize an interaction of “domain specific knowledge, which is acquired through intensive training” with such variation/selection processes.

Poincare (1913) hypothesized five stages of his own creative process: preparation, incubation, intimation, illumination, and verification. The stages of preparation and verification are largely obscure to scientific research as they are carried out over periods of time that extend beyond the times allotted to most experimental protocols (e.g., hours). It is our contention that the cognitive processes between preparation and verification is populated by a blind-variation selective-retention (BVSR), characterized by ideational fluency and originality. This process is accessible to scientific examination, and has been recently examined in a large cohort of normal adult subjects, utilizing a scoring methodology that purports to disentangle fluency from creativity (Silvia et al., 2013). We have further noted the possible roles of excitatory and inhibitory neural processes in modulating this selection–retention process (Jung et al., 2013; Jung, 2014).

The interacting role of excitatory and inhibitory neural processes in creative cognition has been described previously (Heilman et al., 2003; Flaherty, 2005; Abraham and Windmann, 2007). Heilman et al. (2003) was the first to note the importance of frontal lobe interactions with “polymodal and supramodal regions of the temporal and parietal lobes” in divergent thinking, noting that “perhaps these connections are important for *inhibiting* the activated networks that store semantically similar information while *exciting* or activating the semantic conceptual networks that have been only weakly activated or not activated at all. Activation of these remote networks might be important in developing the alternative solutions so important in divergent thinking (page 373).” Flaherty (2005), in her seminal theoretical article regarding neural origins of innovation and creative drive, notes “the appropriate balance between frontal and temporal activity is mediated by mutually inhibitory corticocortical interactions.” Finally, Abraham and Windmann (2007, p. 45) state “The mutual inhibition between frontal language production and temporal language reception has a parallel in the mutually inhibitory effects of idea generation and of assessing what one has produced.” It appears that the field is converging around a bifurcated process involved in producing creative ideas: one involving variation (i.e., cognitive expansion, divergent thinking), and the other involving selection (i.e., constraint of example, usefulness; Abraham and Windmann, 2007; Benedek et al., 2011; Mok, 2014).

We sought to support the equal-odds principle of creative cognition as measured by the fluency–creativity association. Moreover, we sought to link such associations with relevant excitatory (i.e., fluency) and inhibitory (i.e., originality) brain networks as hypothesized previously (Heilman et al., 2003; Flaherty, 2005; Abraham and Windmann, 2007; Jung et al., 2013). We hypothesize that fluency would be associated with creativity as assessed on a measure of divergent thinking, and that fronto-subcortical brain networks would constrain such relationships.

## Materials and Methods

### Sample

This study was conducted according to the principles expressed in the Declaration of Helsinki. The study was approved by the Institutional Review Board of the University of New Mexico (IRB#11-531). All subjects provided written informed consent before collection of samples and subsequent data analysis. Two-hundred and forty-six subjects (127 males; 119 females) between the ages of 16 and 31 (Mean = 21.8; SD = 3.5) were recruited from the University of New Mexico. Subjects were screened by questionnaire to exclude major neurological injury or disease (e.g., traumatic brain injury) and psychiatric disorder (e.g., major depression). All subjects were administered a 4 hours battery of measures including tests of intelligence, personality, and aptitude, and received $100 compensation for their time.

### Behavioral Measures

**FIGURE 1:**
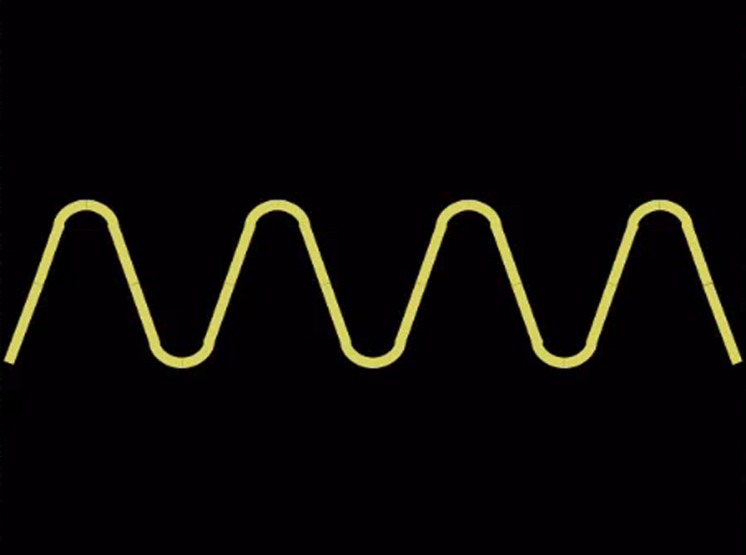
**Example of Foresight figure used to elicit responses from subjects.** Subjects are asked to describe what the figure “makes you think of, looks like, reminds you of, or suggests to you.”

All subjects were administered a broad battery of tests; here we focus on the relationship between measures of divergent thinking (Foresight) and other measures of intelligence (Wechsler Abbreviated Scale of Intelligence II, WASI-II), creativity (Creative Achievement Questionnaire, CAQ), and personality (Big Five Aspect Scale, BFAS) relevant to our hypotheses (Wechsler, 1999; Acton and Schroeder, 2001; Carson et al., 2005; DeYoung et al., 2007). Foresight (reliability = 0.96) measures subjects’ creative thinking ability and comes from the Johnson O’Connor battery of tests of aptitude^1^. Here, subjects are presented with a design and asked to write as many things that the design “makes you think of, looks like, reminds you of, or suggests to you,” in 45 s, over six different designs (**Figure 1**). This measure of divergent thinking over a period of seconds (as opposed to several minutes) is analogous and appropriately comparable to those administered within functional neuroimaging settings.

We used the Consensual Assessment Technique (CAT, Amabile, 1982) to rate subject responses on a scale from 1 to 5 with 1 being least creative and 5 being most creative. Importantly, we used Silvia’s method of “snapshot scoring” wherein all six subject responses were given a single holistic score by three judges (Silvia et al., 2009). This method allows for the extraction of “creative” responses as opposed to merely “unique” responses as is customary in scores of divergent thinking ability (Silvia et al., 2013). The WASI is a standardized measure of intelligence consisting of measures of word knowledge (Vocabulary), verbal associations (Similarities), design construction (Block Design), and non-verbal problem solving (Matrix Reasoning). We did not administer the Vocabulary section of this measure and the Full Scale Intelligence Quotient (FSIQ) was derived from the remaining three subtests. The Creative Achievement Questionnaire (CAQ), has demonstrated adequate reliability and validity as a measure of creative productivity across ten domains including visual arts, music, creative writing, dance, drama, architecture, humor, scientific discovery, invention, and culinary arts (Carson et al., 2005). It has been described as “the most promising” measure of creativity, spanning domain, ability, and conforming to BVSR processes (Simonton, 2012). The BFAS was used to assess personality (DeYoung et al., 2007), particularly the subscale of Openness, which has been consistently related to both divergent thinking and creative cognition (McCrae and Ingraham, 1987).

### Neuroimaging

Structural imaging was obtained using a 3 Tesla Siemens scanner using a 32 channel head coil. We obtained a T1 five echo sagittal MPRAGE sequence (TE = 16.4 ms; 3.5 ms; 5.36 ms; 7.22 ms; 9.08 ms; TR = 2530 ms; voxel size = 1.0 mm × 1.0 × mm 1.0 mm; slices = 192; acquisition time = 6:03). Methods for cortical reconstruction and volumetric segmentation were performed with the FreeSurfer image analysis suite^2^ and are described in detail elsewhere (Fischl et al., 2002, 2004; Han and Fischl, 2007). Briefly, this process includes motion correction and averaging of volumetric T1 weighted images, removal of non-brain tissue, automated Talairach transformation, segmentation of the subcortical white matter and deep gray matter volumetric structures, intensity normalization, tessellation of the gray matter, white matter boundary, automated topology correction, and surface deformation following intensity gradients to optimally place the gray matter/white matter boundary and gray matter/cerebrospinal fluid borders (also known as the pial surface). Thickness measurements were obtained by reconstructing representations of the Gray Matter/White Matter boundary and the pial surface and then calculating the distance between those surfaces at each point across the cortical mantle (Dale et al., 1999). The results of the automatic segmentations were quality controlled and any errors were manually corrected. The cortical thickness parcellation yields 33 measures per hemisphere (i.e., 66 across the surface of the brain) as well as seven subcortical volumes per hemisphere (i.e., fourteen across the brain) including bilateral caudate, putamen, globus pallidus, nucleus accumbens, thalamus, amygdala, and hippocampus (Fischl et al., 2002).

### Analysis

We used bivariate correlation to determine relationships between behavioral measures. Linear regression, controlling for age, sex, handedness, and FSIQ, was used to determine the relationship between measures of fluency (Foresight – total number produced) and Creativity (Foresight – CAT) and measures of cortical thickness and subcortical volumes across the entire brain. CAQ scores were log10 transformed before further analysis as this measure was highly skewed. We did not control for multiple comparisons, although with 66 cortical regions and 14 subcortical regions being analyzed, there are 80 contrasts being made per regression. Given that five in 100 Type I errors are considered to be generally acceptable in research designs, we would expect roughly four regions of 80 to be related to our measures by chance. We have adjusted our significance levels to *P* < 0.005 to account for such possible chance relationships.

## Results

**FIGURE 2:**
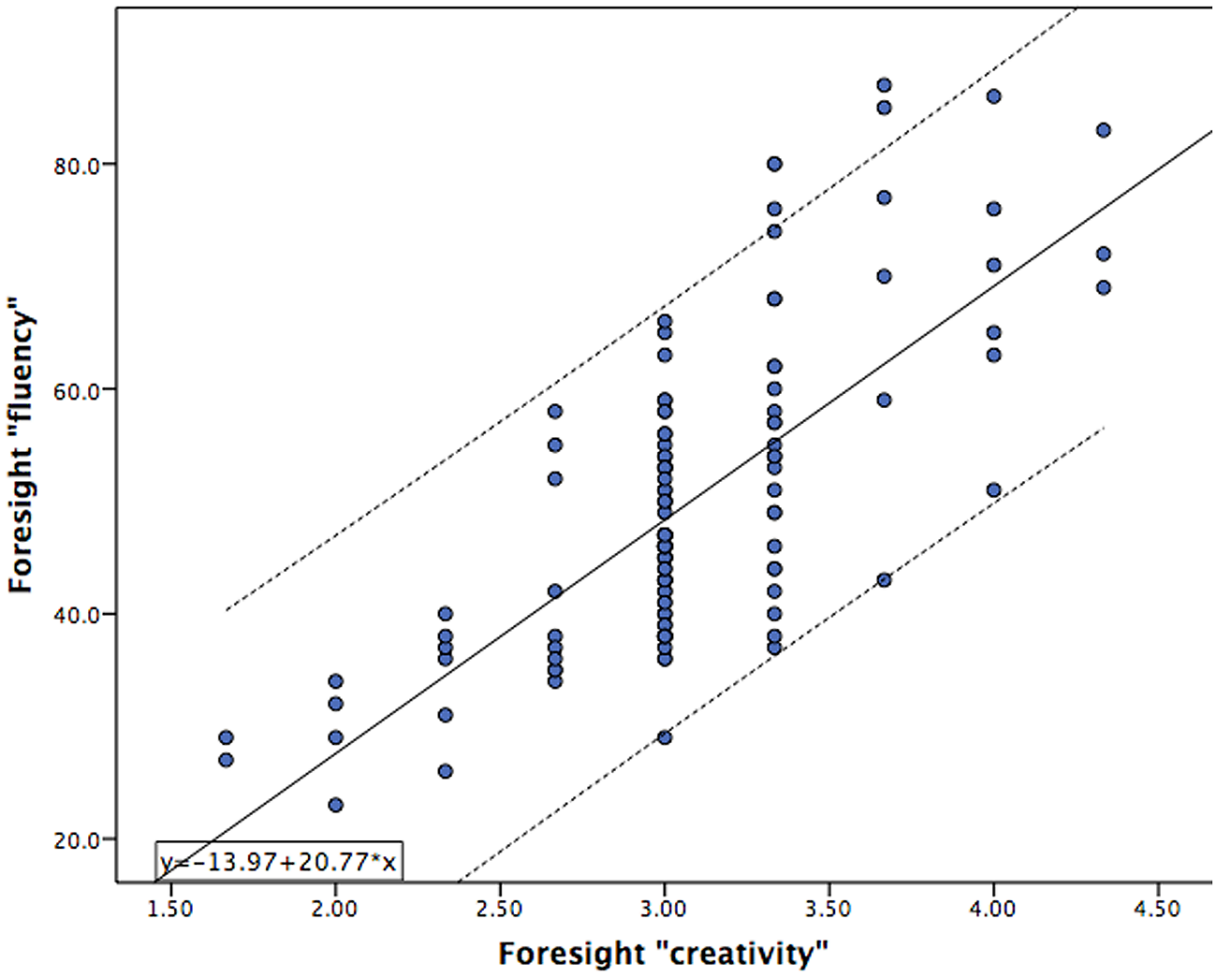
**Scatterplot of “fluency” measures from the Foresight task (y-axis) versus the “creativity” measure (x-axis) obtained from 246 subjects.** Significant overlap in subject scores results in fewer than 246 individual points being observed on the graph.

Subjects were higher than average in terms of intellectual ability (Mean = 111.7; SD = 12.1), as is characteristic of a college cohort, and ranged in IQ from 80 to 153. Creativity scores on the Foresight measure were reliable across the three judges with scores being “good” in terms of internal consistency (Cronbach alpha = 0.76). The relationship between “fluency” and “creativity” scores obtained from the Foresight measure of divergent thinking and other behavioral measures of intelligence, creativity, and personality are presented in **Table 1**. The significant relationship observed between measures of both “fluency” and “creativity” with other proxy measures of creativity, including the CAQ, and Openness, demonstrates convergent validity of this divergent thinking measure. Importantly, “fluency” was highly related to “creativity” in this sample (*r* = 0.73, *p* < 0.001), supporting our hypothesis that ideational quantity is associated with ideational creativity. The relationship between “fluency” and “creativity” on the Foresight measure, across all 246 subjects, is presented in **Figure 2**.

**Table 1:** Bivariate relationships between Foresight measures of fluency and creativity.

	Age	Full scale intelligence quotient	Openness	Creative achievement questionnaire
Fluency	0.09	0.01	0.15	0.17
Creativity	0.07	0.05	0.18^∗^	0.26^∗^

*^∗^p < 0.05.*

**FIGURE 3:**
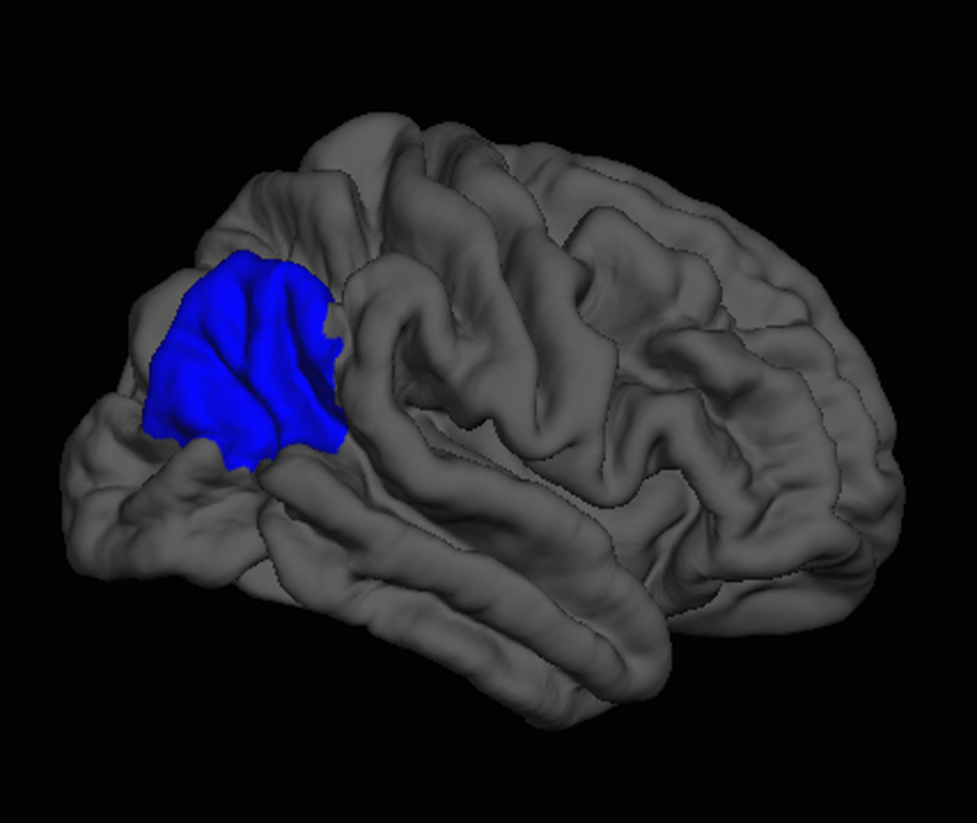
**FreeSurfer rendering of the right hemisphere pial surface with the inferior parietal region indicated in blue, showing decreased cortical thickness in this region associated with increased fluency on the Foresight task**.

**FIGURE 4:**
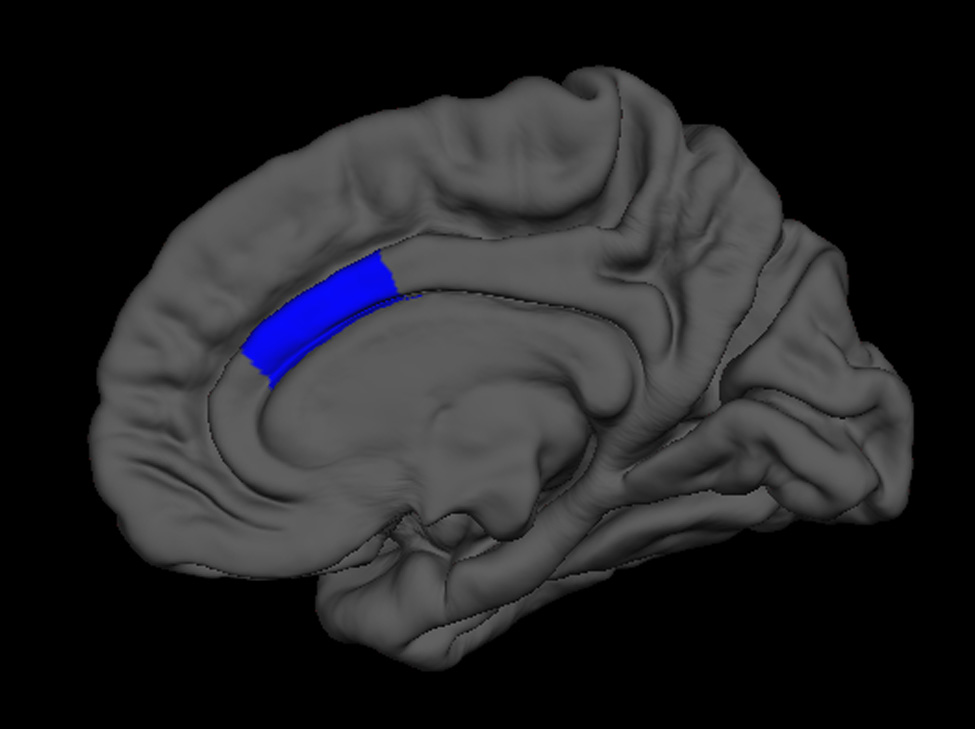
**FreeSurfer rendering of the right hemisphere pial surface with the caudal anterior cingulate indicated in blue, showing decreased cortical thickness in this region associated with increased fluency on the Foresight task**.

Next, we regressed all brain measures of cortical thickness and subcortical volumes against measures of “fluency” and “creativity,” controlling for age, sex, handedness, and FSIQ. “Fluency” was negatively correlated with the volume of the right thalamus (β = -0.24) as well as with the cortical thickness of the right inferior parietal lobe (β = -0.20; **Figure 3**), and caudal anterior cingulate (β = -0.13; **Figure 4**). In contrast, “fluency” was positively correlated with the cortical thickness of the left frontal pole (β = 0.25; *F* = 5.02, *p* < 0.001, *r*^2^ = 0.15).

.

**FIGURE 5:**
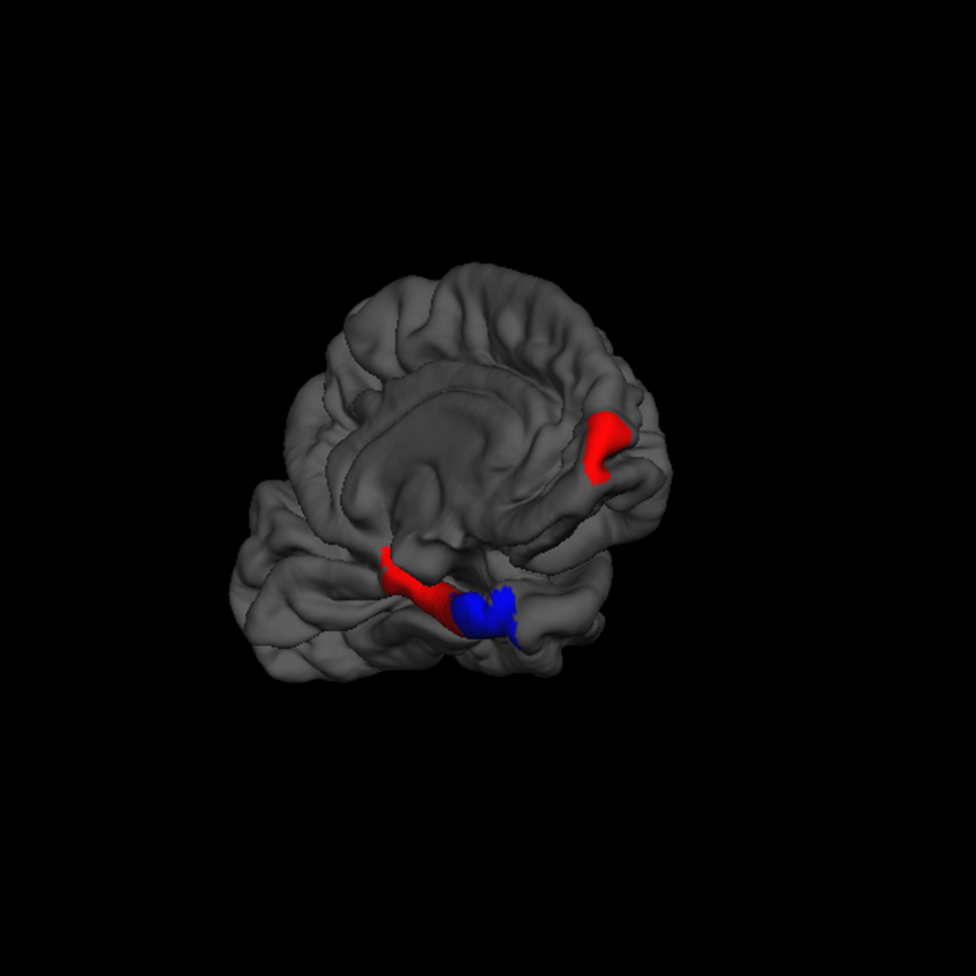
**FreeSurfer rendering of the left hemisphere pial surface with the left frontal pole, parahippocampal gyrus, and entorhinal gyrus indicated (inferior frontal view), showing positive (red) and negative (blue) associations between cortical thickness and increased creativity on the Foresight task**.

“Creativity” was negatively correlated with the volume of the left entorhinal cortex (β = -0.20). In contrast, “creativity” was positively correlated with volume of the left frontal pole (β = 0.17) and left parahippocampal gyrus (β = 0.12; *F* = 3.3, *p* = 0.002, *r*^2^ = 0.09; **Figure 5**).

## Discussion

We found that quantity was associated with quality on measures of divergent thinking customarily associated with creative cognition. Subjects who produced more descriptions of abstract visual designs produced more creative descriptions of the designs as measured by judges who were blind to subject demographics. These results provide compelling support for the equal-odds rule which underlie BVSR theories of creative cognition, and which have been demonstrated repeatedly in Big C cohorts throughout history. Importantly, these results were obtained in a college sample ranging in creative achievement (0–144), and intellectual capacity (80–153), thus spanning the normal ranges of both creative and intellectual abilities. We found that fluency and creativity were highly related to one another when measured using a test of divergent thinking and the consensual assessment technique. Finally, we found that fronto-subcortical brain networks were implicated in performance of both fluency and creativity measures, with a common locus across both measures being the frontal pole.

These results partially replicate our previous findings where we found an inverse relationship between fluency measures of Foresight and volume of the right thalamus in a smaller sample of 107 subjects (Jung et al., 2014). This result is now confirmed in a much larger sample (*N* = 246) that includes those original subjects, and extends these findings into cortical thickness measures. Specifically, we found that a thicker left frontal pole was associated with both higher fluency and higher creativity across subjects. Of interest to this finding, a network that includes the frontal pole and the medial temporal lobes has been implicated in thinking about one’s own future (Okuda et al., 2003). More specific studies undertaken with patients suffering brain lesions have found that frontal pole damage is associated with (1) preference for immediate versus future reward (Bechara et al., 1996), (2) abnormal strategy application (Shallice and Burgess, 1991), and (3) disrupted decision making (Damasio et al., 1991). Researchers have further parcellated the frontal lobe to indicate time valence when thinking about the near future versus far future, and integration with parahippocampal regions when “extracting future prospects” (Okuda et al., 2003). Thus, our results, implicating both left frontopolar and parahippocampal thickening appear to comport well with this particular network implicated in “thinking about the future.” In a large meta-analysis of all functional neuroimaging studies of “episodic future thinking” (EFT) researchers noted the specificity of the medial prefrontal cortex in EFT, indicating its likely role in (1) adaptive decision making processes, (2) the creation of abstract knowledge or schemas, and (3) the integration of novel experiences into pre-existing knowledge networks (Stawarczyk and D’Argembeau, 2015). Our results, demonstrating cortical thickening in the medial frontal lobe, is interpreted to reflect strengthening of neural networks underlying such cognitive processes.

We have previously interpreted thalamic volume decrements and right inferior parietal thinning within a “disinhibitory framework of brain regions associated with increased behavioral output,” and the current findings are consistent with our previous findings implicating thalamic and inferior parietal regions with increased creative capacity (Jung et al., 2013). Other researchers have found lower thalamic dopamine D2 receptor densities to be inversely related to creative cognition in healthy individuals (de Manzano et al., 2010). Importantly, these researchers used a measure of divergent thinking, and the results were related to fluency of uses (as opposed to originality). These authors noted that the thalamus contains the highest level of dopamine D2 receptors in the brain, and that decreased D2 binding has been linked with decreased “filtering and autoregulation of information flow,” as well as decreased inhibition of prefrontal pyramidal neurons (Seamans and Yang, 2004; Trantham-Davidson et al., 2004). They refer to this decreased inhibition as producing a “creative bias” which benefits tasks requiring continuous generation and increases fluency and flexibility of associations. Our results, reflecting both lower thalamic and anterior cingulate volume (Bush et al., 2000) would be broadly consistent with this hypothesis, and provides further neurobiological support to better explain the equal-odds phenomenon underlying variation/selection mechanisms associated with creativity.

There are several limitations to our approach. First, we utilized a relatively young, healthy sample, and whether our results would generalization to older populations and/or clinical samples is unknown. Second, we are making inferences regarding brain function in spite of using measures of brain structure. These inferences may or may not be correct, although some studies suggest correspondence between structure and function (Segall et al., 2012). Our measure of divergent thinking is not one commonly used in the creativity literature, although it was found to have high reliability, as well as correspondence to other measures commonly used as proxy measures (e.g., CAQ, Openness) for creativity. Finally, we have not conducted full Bonferroni correction for all possible multiple corrections (which would increase Type II error), but have adopted an intermediate approach of adjusting our significance level to *p* < 0.005 to account for the 80 contrasts being made per regression (leaving possible Type I error). This balance between Type I and Type II error was seen as appropriate for this exploratory study. Future studies using broader samples comprised of both older and younger subjects, using other well-validated measures of fluency-originality, and exploiting multimodal neuroimaging measures [e.g., structural Magnetic Resonance Imaging (MRI), functional MRI, diffusion tensor imaging] would help significantly to support these findings and to implicate particular brain networks associated with BVSR.

This study supports the notion that BVSR is a central component of creative cognition, working via an equal-odds rule, wherein higher output of ideas is associated with higher likelihood of creative ideas. This paradigm is amenable to both psychological and neuroscientific manipulation to determine the interaction of fluency–creativity relationships with other cognitive components hypothesized to be relevant to creative cognition (e.g., cognitive control, flexibility, etc.). Our results demonstrate key nodes within the brain, including the right thalamus, right caudal anterior cingulate, left medial temporal lobe, left medial frontal cortex, and right temporo-parietal junction that constrain both fluency and originality in a manner that would suggest mutually inhibitory network interactions constraining both variation and selection processes. Specific nodes (e.g., medial fronto-temporal cortices) within this network have been implicated in highly adaptive human cognitive processes including EFT and extracting future prospects, while other regions (e.g., thalamus, anterior cingulate) have been implicated in modulating the fluency and flexibility of ongoing cognition. Future research will be critical in determining the specific roles that these structures play in the interactions between broad cognitive networks that have been implicated in creative cognition (e.g., default mode network).

In summary, early theories regarding creative cognition have broadly implicated fronto-temporal and fronto-subcortical networks that operate in “mutually inhibitory” balance that, when disease (Miller et al., 1998) or lesion (Shamay-Tsoory et al., 2011; Abraham et al., 2012) disrupt this balance, can result in greater creative drive and/or novelty generation (Flaherty, 2005). More current theories implicate an interaction between broad networks of the brain including the default, executive control, and salience, which interact in service of variation and selection tasks organized around adaptive behavior (Jung et al., 2013; Beaty, 2015; Liu et al., 2015). Research is converging around two main aspects of creative cognition involving variation (i.e., divergence, fluency, elaboration) on the one hand and selection (i.e., convergence, usefulness, constraint) broadly conforming to notions of BVSR (Campbell, 1960). This “variation-selection” model is evolutionarily sound, conforms to humans and other living species, and produces adaptive behaviors within “design space” (Dennett, 1996).

## Conflict of Interest Statement

The authors declare that the research was conducted in the absence of any commercial or financial relationships that could be construed as a potential conflict of interest.

## Footnotes

1 http://www.jocrf.org

2 http://surfer.nmr.mgh.harvard.edu/
